# Supplementary material for: Transcriptomic and Proteomic Analyses Show Minimal Role for ST2 on MCPT5‐Expressing Mast Cells in Primary Heligmosomoides polygyrus bakeri Infection
Source: Parasite Immunol. 2026 Mar 30;48(4):e70076. doi: 10.1111/pim.70076 (PMC13034402; doi:10.1111/pim.70076)
Supplement: Supplementary file 4 — Figure S1: Flow cytometric detection of FcεRI changes across Hpb infection due to IgE‐binding. WT C57BL/6J mice were infected with 200 L3 Hpb larvae by oral gavage. Separate cohorts of mice were culled at indicated days of Hpb infection. Peritoneal lavage (PL) was prepared for flow cytometry to assess mast cell FcεRI expression (geometric mean fluorescence intensity [gMFI] normalised to gMFI in naïve mice) (A). Serum levels of total IgE (B) were also measured which showed a negative correlation with mast cell FcεRI gMFI (C). Data in (A–C) is representative of 2 experiments, each with n = 6/group. Mice were 8–12 weeks old, with only male WT C57BL/6J mice used. Error bars show mean ± SEM. Flow cytometric analysis of PL mast cells also showed a negative correlation between detection of surface FcεRI and IgE, with representative samples in naïve, Hpb D7 and Hpb D28 mice shown (D). As such, gating strategy of mast cells was updated to include IgE+ cells (E). [file PIM-48-e70076-s003.pptx]

## Slide 1
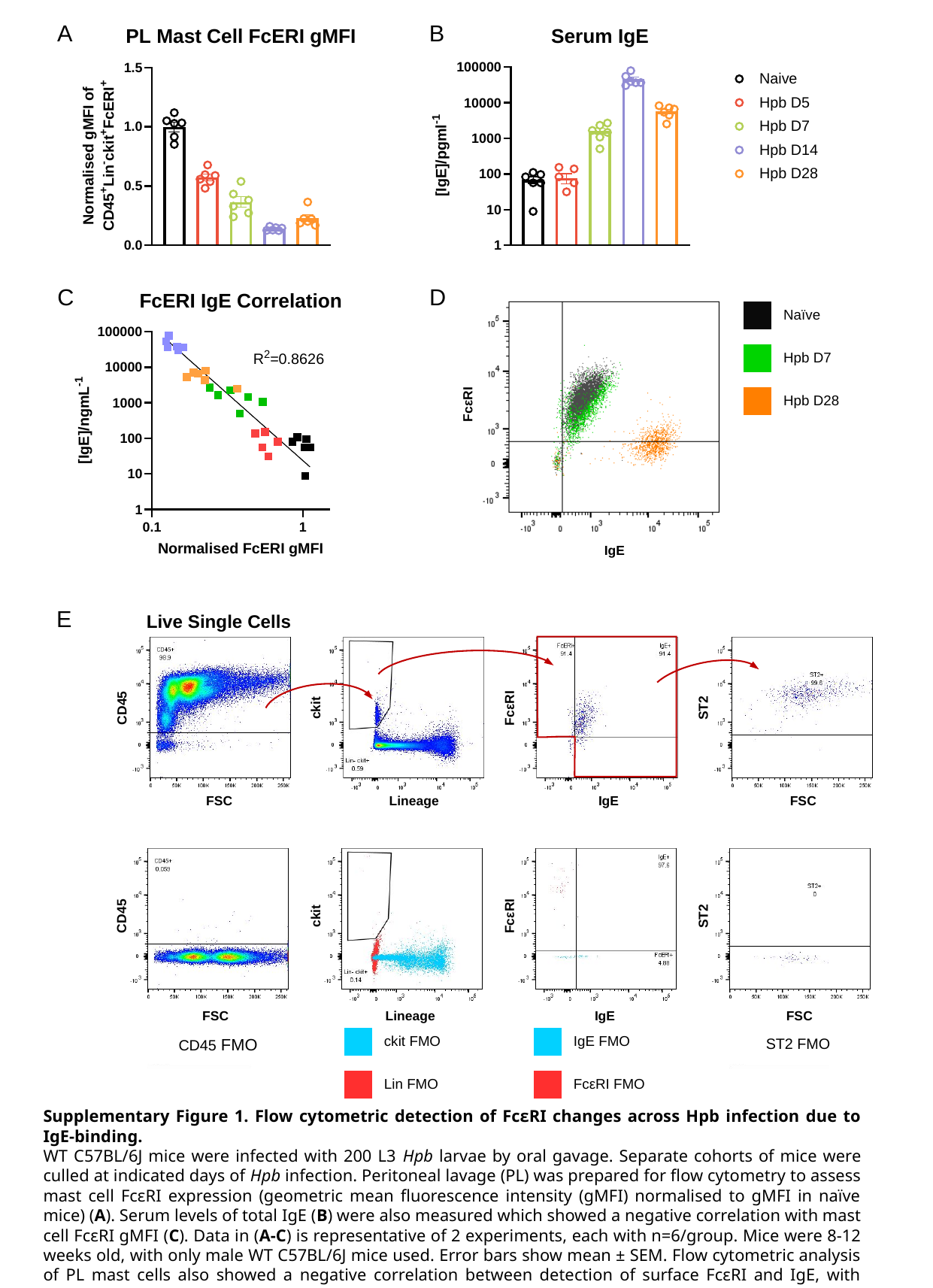

A
B
C
D
FcεRI
IgE
| | Naïve |
| --- | --- |
| | |
| | Hpb D7 |
| | |
| | Hpb D28 |
E
Live Single Cells
CD45
ckit
FcεRI
ST2
FSC
Lineage
IgE
FSC
CD45
ckit
FcεRI
ST2
FSC
Lineage
IgE
FSC
| | IgE FMO |
| --- | --- |
| | |
| | FcεRI FMO |
| | ckit FMO |
| --- | --- |
| | |
| | Lin FMO |
CD45 FMO
ST2 FMO
Supplementary Figure 1. Flow cytometric detection of FcεRI changes across Hpb infection due to IgE-binding.
WT C57BL/6J mice were infected with 200 L3 Hpb larvae by oral gavage. Separate cohorts of mice were culled at indicated days of Hpb infection. Peritoneal lavage (PL) was prepared for flow cytometry to assess mast cell FcεRI expression (geometric mean fluorescence intensity (gMFI) normalised to gMFI in naïve mice) (A). Serum levels of total IgE (B) were also measured which showed a negative correlation with mast cell FcεRI gMFI (C). Data in (A-C) is representative of 2 experiments, each with n=6/group. Mice were 8-12 weeks old, with only male WT C57BL/6J mice used. Error bars show mean ± SEM. Flow cytometric analysis of PL mast cells also showed a negative correlation between detection of surface FcεRI and IgE, with representative samples in naïve, Hpb D7 and Hpb D28 mice shown (D). As such, gating strategy of mast cells was updated to include IgE+ cells (E).
